# The effect of object perception on event integration and segregation

**DOI:** 10.3758/s13414-024-02922-6

**Published:** 2024-09-18

**Authors:** Gülşen Balta, Elkan G. Akyürek

**Affiliations:** 1 Department of Psychology, Experimental Psychology, University of Groningen, Grote Kruisstraat 2/1, 9712 TS Groningen, The Netherlands; 2 Research School of Behavioural and Cognitive Neurosciences, University of Groningen, Groningen, The Netherlands

**Keywords:** Object perception, Event perception, Temporal integration, Selective attention

## Abstract

The perceptual system must integrate information from various points in time and space to interpret continuous sensory input into meaningful units, such as visual objects or events. To explore the relationship between the perception of spatial objects and temporal events, we modified the missing element task, a typical temporal integration task, by inserting a simple spatial object. The aim was to determine whether the perceptual processing of the object would have an impact on the frequency of temporal integration and segregation. Temporal integration was most successful when the missing element was located within the object, less successful when there was no object, and least successful when the missing element appeared outside the object. The advantage of the location of the missing element within the object was observed at display durations from 30 ms to 150 ms. Interestingly, the object provided the same benefit for integration and segregation despite their opposing perceptual demands. This study demonstrates the relationship that exists between the processing of temporal events and spatial objects, and shows how such spatial information can facilitate temporal integration.

**Supplementary Information:**

The online version contains supplementary material available at 10.3758/s13414-024-02922-6.

## Introduction

The visual environment we are surrounded by provides a constant stream of information, across both space and time. For the perceptual system to interpret these continuous sensory inputs, from the most basic visual elements, such as color or shape, to the complex visual scene, they must be ordered and organized. The most fundamental form of this organization is the segmentation or grouping of continuous visual inputs into discrete meaningful units, such as an object in space or an event in time. However, it is challenging to fully understand how our perceptual system breaks down our visual surroundings into these units, without also considering possible spatiotemporal interactions.

Typically, as researchers consider how visual perception is organized, they primarily consider how visual inputs appear and how they are positioned in space, whereby perceptual objects are usually defined in terms of their spatial arrangement of subcomponents (e.g., Hoffman & Richards, 1984; Marr, 1982; Kubovy & Pomerantz, 1981). However, in many real-world situations, an object does not always reach our vision all at once, due to unstable visual inputs and interruptions in the flow of visual information caused by blinks, occlusions, and saccades. Despite this instability of visual information, the ability of our brain to achieve integrity suggests that information is grouped and structured both spatially and temporally. For example, an object moving behind a smaller occluding surface will be perceived as a single moving unit, rather than as individual units, corresponding to each part that is visible at any given time. This suggests that two processes are needed: Components of each object must be detected by spatial grouping of their constituents, and those components that fit together must be integrated accordingly over time in order to produce this consistent impression. To achieve the desired percept as the scene unfolds, we might infer from this illustration that such integration of visual data in time and space needs to happen nearly simultaneously, in interactive fashion. The purpose of the current study was to assess the role of spatial (object-related) properties on the perception of temporal events.

### Perceptual objects

Gestalt psychologists, who were some of the first to attempt to study perceptual organization, developed rules to describe how the perceptual system structures perceptual environments and allows us to perceive objects at a basic level. Examples include the rule of similarity, which holds that when elements are similar to each other (such as in color, size, or orientation) they tend to be integrated into groups, and the rule of proximity, which states that nearby elements are more likely to be grouped together than those that are far apart (Kubovy et al., 1998; Wertheimer, 1924/1950). There is considerable evidence that such object-based organization can be done automatically and without requiring deliberate effort (Duncan, 1984; Egeth & Yantis, 1997; Lamy & Tsal, 2001). Perceptual grouping might indeed precede, and occur independently of, the allocation of attentional resources, and grouping effects can arise regardless of whether the feature of grouping is relevant to the task or not (Driver, 1995; Duncan, 1984; Russell & Driver, 2005; Shomstein et al., 2010). Nevertheless, contextual information can aid the allocation of attention to objects in natural scenes (Torralba et al., 2006). Furthermore, some studies focusing on specific Gestalt grouping rules have indicated a role for attention in perceptual grouping, indicating that not all kinds of groupings emerge automatically (Ben-Av et al., 1992; Houtkamp et al., 2003; Kimchi, 2009; Kimchi & Razpurker-Apfeld, 2004; Mack et al., 1992; Roelfsema et al., 2010; Scholte et al., 2001). For instance, according to Trick and Enns (1997), clustering does not require attention, but shape construction does.

A related perspective contends that perceiving an object or grouping is not a single integrated process but rather a series of phases, each of which demands their own level of attention (Kimchi & Razpurker-Apfeld, 2004). In line with this belief, Treisman’s influential feature integration theory (FIT) proposes that basic, disparate elements of visual scenes (such as colors, orientations, or luminance levels) reach our perception first, after which attention has to be deployed serially to integrate or group them over space to form object and event representations (Treisman, 1996; Treisman & Gelade, 1980). In this conception, focal attention serves as the “glue” that connects simple features and puts previously disparate parameters together to produce unified objects, implying that there are multiple steps involved in object perception. In the classic model of visual search by Wolfe (1994, 2021), object identification is similarly conceived of as a second, capacity-limited stage. In the model of spatial attention by Itti and Koch (2000), object identification is altogether relegated to a separate process.

In addition to the role of attention in the formation of a perceptual object, the perceptual object or organization itself also impacts how attention is distributed over the visual scene. Two main hypotheses on where the focus of attention might be directed—to a spatial location or to an object—are discussed in the extensive literature on visual attention. Earlier studies on selective attention assumed that visual attention moves across the visual field like a spotlight and selects images based on their spatial location (e.g., Posner et al., 1980). However, subsequent research has revealed that space is not the only frame of reference for selective attention. Duncan (1984) demonstrated that even when the spatial location is held constant, attending to multiple objects incurs a cost, compared with attending to a single object. Egly et al. (1994) further confirmed that both factors are significant. In their two-rectangle paradigm, attention was manipulated by a spatial cue, and the cost of switching attention from the cued position to an uncued position was greater between the two rectangle objects than within each object, despite both uncued locations being at an equal distance from the cued location, indicating attention has an object-based component. Finally, to measure how attention is captured by a perceptual object, Kimchi et al. (2016) conducted serial experiments on the involvement of spatial components, different types of Gestalt organization, and strength of perceptual organization. Their findings indicated that a perceptual object immediately draws attention, in a completely stimulus-driven fashion. It was established that a spatial component plays a role in the automatic capture of attention, that collinearity and proximity of the Gestalt factors facilitated this attentional capture, and that this attentional capture is determined by the level of perceptual organization. The authors therefore interpreted these results to indicate that perceptual organization affects how attention is automatically distributed.

### Perceptual events

As stated above, visual input can also be reliably and meaningfully separated into temporal segments and events, just as visual scenes are spatially subdivided into objects. A temporal segment that is thus created has been referred to as a perceptual moment (Allport, 1968; Efron, 1967). It was hypothesized that continuous visual stimuli are divided into discrete chunks or segments, each of which lasts for about 100 ms. A single appearance is then produced by perceptually integrating the visual inputs that arrive at the same temporal moment. This conceptualization views the perceptual moment as a temporal window that contains information constantly for its entire duration, with old stimuli disappearing as new ones come into the window. This reasoning implies that two stimuli that are separated by less than 100 ms should be combined into a single event.

However, it turned out that if the onset asynchrony between successive stimuli is kept constant, while the duration of the leading stimulus is increased, the integration of subsequent stimuli declines (Coltheart, 1980; Di Lollo, 1980). This finding is hard to account for in terms of a perceptual moment. Therefore, the integration of successive visual input may be better explained by another mechanism—namely, visible persistence. Visible persistence entails that after the onset of a visual stimulus, a perceptual representation is activated in the visual system that persists for a short period of time (about 130 ms). This persistence period allows the integration of the first stimulus with the following one that arrives during this period. More specifically, the temporal correlation between the persisting representations of successive stimuli predicts the likelihood of integration (Coltheart, 1980; Di Lollo, 1980; Di Lollo et al., 1994).

The integration of temporal information is also impacted by the spatial arrangement of visual inputs. It has been found that as the spatial proximity between subsequent segments in a stimulus sequence decreases, temporal integration progressively shortens, which was thought to be a mechanism to prevent motion smear (Burr, 1980; Di Lollo & Hogben, 1985; Farrell, 1984). Similarly, Di Lollo and Hogben (1987) showed that the integration of two subsequent dot arrays was noticeably impaired with increased interdot separation. A study by Hermens et al. (2009) implemented feature fusion paradigms that entailed the combination of a vernier and an anti-vernier, to investigate the relationships between spatial and temporal grouping. They found that integration of the anti-vernier with the prior vernier was hindered when it was grouped with the neighboring anti-vernier in terms of both spatial proximity and similarity. Another illustration is a study on temporal order judgment by Nicol and Shore (2007), who found that grouping two successive stimuli into a single perceptual object, rather than recognizing them as two discrete objects, resulted in worse order judgment performance.

Finally, as in object perception, the availability of attention also influences temporal integration (Hochmitz et al., 2021; Sharp et al., 2018; Visser & Enns, 2001; Yeshurun & Levy, 2003; Yeshurun & Marom, 2008; but see also Balta et al., 2020). The first line of evidence for a link between attention and temporal integration comes from studies investigating the effect of focused attention on the perceived length of a stimulus (Enns et al., 1999; Mattes & Ulrich, 1998). In these studies, the participants’ perceptions of time were assessed for brief flashes in either attended or unattended locations, and the same flashes were perceived to last longer in attended sites than they did in unattended sites. These findings imply that temporal integration can be improved by attention since attention lengthens the perceived duration of the stimulus and allows a longer perception time for a stimulus to integrate with a subsequent input.

In a study of Visser and Enns (2001), this relationship between attention and temporal integration was more explicitly tested. They used the attentional blink paradigm to modulate temporal attention and demonstrated that integration was enhanced when attention was more readily available. Akyürek and van Asselt (2015) obtained further evidence for the positive correlation between attention and integration. These authors used a spatial cue to measure the effect of attention on color fusion, which is thought to be one of the earliest forms of temporal integration. When attention was enhanced by giving a valid cue beforehand, the subsequent presentation of two colored squares in quick succession was more likely to be perceived as a single fused color, indicating that temporal integration was facilitated by attention. Another study investigating the effect of spatial attention on temporal integration using a missing element task was conducted by Sharp et al. (2018). In their study, endogenous attention was manipulated using location cues and it was found that the endogenous deployment of spatial attention prolonged the temporal integration window. Similarly, a recent study of Hochmitz et al. (2021) demonstrated that endogenous attention extends the time available for information to integrate in the Ternus display when examining the effect of endogenous attention on motion perception. Taken together, these findings indicate that attention is also an important factor in temporal integration.

### The present study

As we have argued, although spatial and temporal aspects of perception are often studied independently, it is also important to take into account how spatial and temporal integration processes interact. The current study investigated how spatial and temporal factors interact while we integrate simple features into a single event representation. In particular, we examined how visual temporal integration is affected by spatial object perception, across different stimulus durations. We used the Missing Element Task (MET; Akyürek et al., 2010; Hogben & Di Lollo, 1974) to measure temporal integration and segregation performance. In this paradigm, an array of small squares is presented in two brief successive displays, with half of the items presented in one display and the other half—minus one item—in the other display. Participants are instructed to report the location of the missing item, which is the single square in a grid where no item is presented in either the first or the second display. The task is virtually impossible to carry out from memory, and can be achieved only if the two displays are temporally integrated during perception (Di Lollo, 1980; Irwin & Yeomans, 1986). Critically, a subset of the items in the stimulus sequence was grouped into rectangular-shaped objects, by presenting them in different colors. We were interested to assess whether the appearance and perception of the object would cause (attention-mediated) effects on the rate of temporal integration, and conversely, segregation. Specifically, we hypothesized that temporal integration would be facilitated at locations inside the object, as compared with locations outside the object, and as compared with trials without an object, while the opposite pattern was expected for segregation.

## Experiment 1

In Experiment 1, we focused on integration, hypothesizing that it would benefit from the presence of an object, in particular when the location of the object and missing element coincided. Conversely, we expected lower integration performance when the object and missing element did not coincide. Performance when there was no object present should fall in between these two cases.

### Method

#### Participants

Forty-one participants (seven male) participated in the study in exchange for either course credit or monetary compensation. All participants reported normal or corrected-to-normal visual acuity and no color blindness. Informed consent was obtained in writing prior to their participation. The study was carried out in accordance with the Helsinki Declaration and approved by the local Ethical Committee at the University of Groningen (approval number 2122-S-0086). The mean age was 20.35 years (range: 18–33), and eight participants were excluded from the analysis due to low performance, which was defined a priori as less than 20% overall accuracy.

#### Apparatus and stimuli

The experiment was programmed in Open Sesame 3.2 (Mathôt et al., 2012) and run on the Microsoft Windows 10 operating system with the Expyriment back end (Krause & Lindemann, 2014). During the experiment, participants were seated individually in a sound-attenuated cabin with dimmed lighting. The viewing distance was set to be approximately 60 cm from the screen, and a regular USB mouse was used to record the responses. Stimuli were displayed on a 19-in. CRT screen with a refresh rate of 100 Hz and a resolution of 800 by 600 pixels in 16-bit color. Experimental stimuli were 49 squares that were arranged in a grid of 7 by 7 positions, and the size of the entire grid was 140 by 140 pixels (6.72**°** by 6.72**°** of visual angle). The squares were either all presented in red (RGB 255, 0, 0) or in black (RGB 0, 0, 0), or 12 of them, arranged in a 3 by 4 or 4 by 3 grid with a 60 by 80 pixel size (2.88**°** by 3.84**°** of visual angle), were presented in one color and the rest in the other. The size of a single square was 10 by 10 pixels (0.48**°** by 0.48° of visual angle) inserted in the center of an invisible square of 20 by 20 pixels (0.97**°** by 0.97° of visual angle). All stimuli were shown across the two successive target displays and were centered on the screen while maintaining a uniform white background. The response screen consisted of 49 black outlined squares, again arranged in a grid of 7 by 7 positions in the center of the screen.

#### Procedure and design

**Fig. 1:**
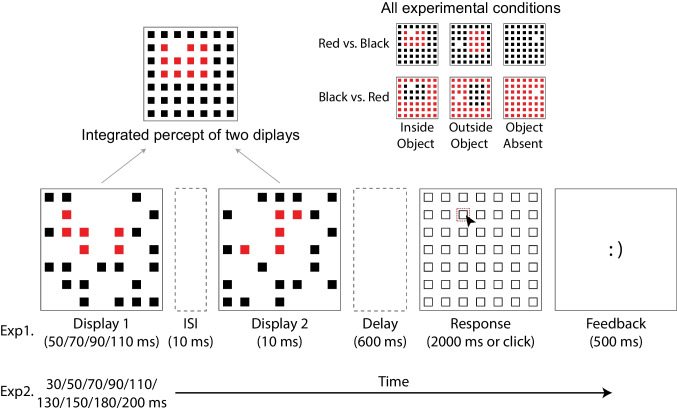
Schematic representation of a single trial used in Experiment 1 and Experiment 2. Two successive arrays of small squares were shown, arranged in a 7 by 7 grid in the center of the display. The squares were either all presented in red or in black, or 12 of them, arranged in a 3 by 4 or 4 by 3 grid, were presented in one color and the rest in the other (all possible combinations of conditions shown in the upper right). In the first display, 24 out of 49 possible squares were drawn, after 10 ms of ISI, the second set of 24 squares was shown. Following a 600-ms blank interval, the response screen was displayed, and participants were asked to indicate the location of the 49^th^ square that was not shown (in red dashed outline for illustration purposes). Lastly, a brief feedback screen was provided. (Color figure online)

The experimental trials started with the presentation of a blank display for 600 ms, followed by the first stimulus array that lasted for variable durations (50, 70, 90, or 110 ms). Twenty-four randomly chosen squares were shown in the first stimulus display, and after 10 ms of ISI, the second stimulus display that contained 24 squares at nonoverlapping grid locations was presented for 10 ms. The merging of these two stimulus displays formed a square stimulus grid. If there was no object present in the trial, all squares were shown in the same color (black or red). If there was one, this entailed that 12 of the squares were colored differently to form a rectangular-shaped object (red vs. black or black vs. red; object layout either horizontal or vertical). Across two stimulus displays, 48 out of 49 squares were presented in total, with one of the grid locations being left empty (see Fig. 1). The missing squares in all conditions, and rectangular objects in object-present conditions, were always presented at random locations in the inner 5 by 5 squares within the larger 7 by 7 grid. In object-present trials, the missing square appeared either in a square position inside the object (in-object) or in a square position outside the object in the inner grid (out-object). In object-absent trials, the missing square appeared in any square position in the inner grid, without there being an object present also.

Afterwards, following a 600-ms blank interval, the response screen was displayed for 2,000 ms or until the participant responded. The participant’s task was to identify the location that had not been filled in either stimulus display. They used the left mouse button to register their responses by clicking on the location of the missing square. Lastly, as is typical in METs, a feedback screen was shown following the response and lasting 500 ms. If participants had the correct response, they saw a happy emoticon [:)], whereas if they had responded incorrectly, they saw a sad emoticon [:(]. Trial-wise feedback was given to increase task engagement and to motivate participants to perform accurately.

Participants completed a total of 1,680 trials, divided into four blocks, which were preceded by 40 practice trials that were discarded from the analysis. The trials were equally divided into three experimental conditions (in-object, out-object, and object-absent). The duration of the first display, the three object conditions, and the object orientation were all randomized within each block. The color of the object and background grid were kept the same in the first two blocks (e.g., red object vs. black grid for trials with the object, and only black grid for trials without the object), and then the colors were switched the other way around in the last two blocks. Color order was counterbalanced across subjects.

Two-way repeated-measures analyses of variance (ANOVAs) and paired-sample *t* tests were used to make statistical comparisons between experimental conditions. The two independent variables were duration (50, 70, 90, and 110 ms) and object condition (in-object, out-object, and no-object), and the dependent variable was the accuracy rate. JASP (Version 0.16.3; JASP Team, 2022) was used for statistical analysis. When the sphericity assumption was violated, the degrees of freedom were adjusted using Greenhouse–Geisser epsilon correction. The Bonferroni correction was applied to adjust the alpha values in all post hoc comparisons. The Supplementary Materials include further analyses and descriptives that were added upon suggestions made by reviewers.

### Results

**Fig. 2:**
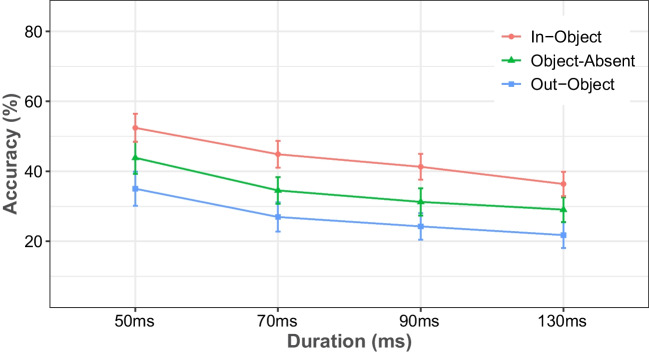
The percentage of correct responses in Experiment 1, plotted as a function of the duration of the first stimulus display. The red line represents the condition where the target was inside the rectangular object, the blue line represents the condition where the missing square was outside the object, and the green line represents the condition where no object was presented. Error bars represent 95% confidence intervals. (Color figure online)

Figure 2 shows the percentage of correct responses as a function of the first stimulus duration separately for each object condition. The analysis yielded significant main effects of duration, *F*(1.81, 58.12) = 114.488, *MSE* = 0.652, *p* < .001, η_p_^2^ = 0.3, and object, *F*(2, 64) = 260.118, *MSE* = 0.004, *p* < .001, η_p_^2^ = 0.469, whereas there was no significant effect for the interaction of the two factors. As is typical of performance in the MET, integration performance decreased with increasing first stimulus duration (43.8% at 50 ms, 35.5% at 70 ms, 32.3% at 90 ms, and 29.1% at 110 ms), and this pattern was basically the same for all object conditions. Furthermore, mean performance was highest for the in-object condition (43.8%), intermediate for the object-absent condition (34.7%), and lowest for the out-object condition (27%). Post hoc *t* tests demonstrated that the difference in performance was significant between in-object and object-absent, *t*(32) = 12.335, *SE* = 0.007, *p* < .001, *d* = 0.78, between in-object and out-object, *t*(32) = 22.728, *SE* = 0.007, *p* < .001, *d* = 1.441, and between object-absent and out-object, *t*(32) = 10.448, *SE* = 0.007, *p* < .001, *d* = 0.661.

**Fig. 3:**
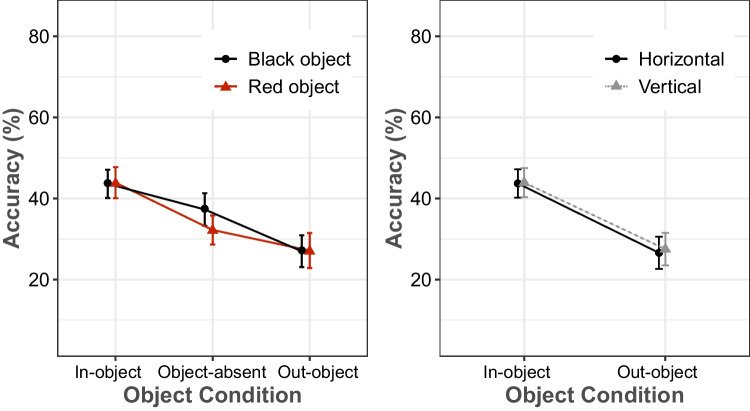
Mean percentage of integration performance as a function of object condition, separately for the two different color conditions of the object and grid (left) and for the two object orientations (right) in Experiment 1. Error bars represent 95% confidence intervals. (Color figure online)

We also performed two additional analyses to examine whether the color of the object and grid and the orientation of the object affected integration performance. The results of these analysis are shown in Figs. 3a and b. A repeated-measures ANOVA of integration performance, with the factors of object color (red and black) and object condition (in-object, out-object, and object-absent), revealed a main effect of object condition, *F*(2, 64) = 264.109, *MSE* = 0.002, *p* < .001, η_p_^2^ = 0.892, and its interaction with object color, *F*(2, 64) = 13.595, *MSE* < 0.001, *p* < .001, η_p_^2^ = 0.298. There was also a marginal main effect of object color, *F*(1, 32) = 3.628, MSE = 0.005, *p* = .066, η_p_^2^ = 0.102. Overall performance averaged 34.3% in red color and 36.1% in black color. Pairwise comparisons showed that the difference between red and black color was significant (32.2% vs. 37.3%) only in the object-absent condition, *t*(32) = −4.429, *SE* = 0.011, *p* < .001, *d* = 0.447. In a further analysis, a repeated-measures ANOVA on integration performance, with the factors of object orientation (horizontal and vertical) and object condition (in-object and out-object), revealed a main effect of object condition, *F*(1, 32) = 453.870, *MSE* = 0.002, *p* < .001, η_p_^2^ = 0.934, but failed to reveal any significant effects for object orientation or for the interaction of the two factors.

The findings of Experiment 1 demonstrated that, for all tested durations, the location of the missing square was best identified when it was inside the object, less well identified in a grid without an object, and poorest when it was outside of the object, implying a basic relation between temporal and spatial processes. Furthermore, in the object-present conditions, the color changes between the rectangular object and the rest of the grid (red object inserted black grid or vice versa) had no effect on integration performance. Similarly, object orientation (vertical or horizontal) had no effect on integration performance. Overall, color only had an effect on integration performance in the object-absent condition. This difference could be explained by relative stimulus contrast, as the white target location was on all-black array rather than an all-red array. Related findings were reported in a study by Akyürek and Meijerink (2012). They assessed the detection of a missing (i.e., white) square and a red square in a single stimulus display condition (MET without integration). The results showed that the accuracy of finding the missing square was significantly greater than the accuracy of finding the red square. It was suggested that the reason for the difference was that the contrast of the missing square against the black squares surrounding it in the array was higher than that of the red square; a similar logic may apply here.

## Experiment 2

The purpose of Experiment 2 was to assess whether the main effects of the object conditions observed in Experiment 1 would generalize across a broader temporal window, extending into durations at which integration either is very likely (as short durations) or rather unlikely (at longer durations).

### Method

#### Participants

Thirty new participants (six males; mean age = 20.17 years; range: 18–29) participated in this experiment. Because of poor overall performance, data from one participant were omitted from the study.

#### Apparatus, stimuli, procedure, and design

The experimental procedure and design were identical to those of Experiment 1, with the exception of the following modifications. More durations were used for the first stimulus display (30, 50, 70, 90, 110, 130, 150, 180, and 200 ms). Since there was no difference observed for color changes between the object and background grid in the object conditions, the color for the entire experiment was kept the same; a red object and a black background grid was used. The total number of experimental trials was 1,512, preceded by 54 practice trials.

### Results

**Fig. 4:**
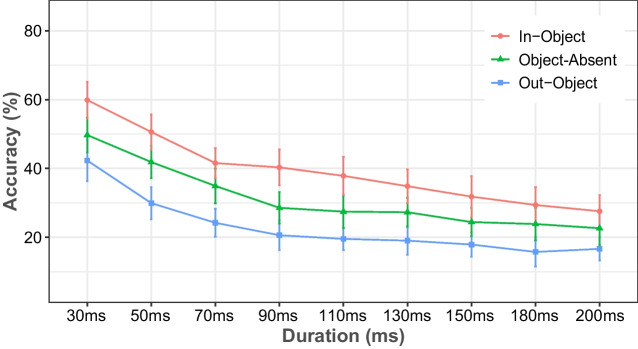
The percentage of correct responses in Experiment 2, plotted as a function of the duration of the first stimulus display, separately for each object condition. Error bars represent 95% confidence intervals

The results of Experiment 2 are shown in Fig. 4. Two-way repeated measure ANOVA revealed that there was both a main effect of duration, *F*(2.89, 81.09) = 93.221, *MSE* = 0.021, *p* < .001, η_p_^2^ = 0.769, and object condition, *F*(1.63, 45.50) = 198.934, *MSE* = 0.011, *p* < .001, η_p_^2^ = 0.877, as well as their interaction, *F*(16, 448) = 2.403, *MSE* = 0.004, *p* = .002, η_p_^2^ = 0.079. Post hoc tests showed that integration was greater for the in-object (38.5%) than for either the object-absent (30.3%), *t*(28) = 9.994, *SE* = 0.008, *p* < .001, *d* = 0.619, or the out-object conditions (22.1%), *t*(28) =19.947, *SE* = 0.008, *p* < .001, *d* = 1.235. There was also a significant difference between the object-absent and out-object conditions, *t*(28) = 9.952, *SE* = 0.008, *p* < .001, *d* = 0.616.

As the duration of the first stimulus increased from 30 ms to 90 ms, overall integration performance fell markedly (49.3%, 39.7%, 32.7%, and 29% at 30 ms, 50 ms, 70 ms, and 90 ms, respectively), but it did not change much beyond 90 ms (27.6%, 26.3%, 24.2%, 22.5%, and 21.8% at 110 ms, 130 ms, 150 ms, 180 ms, and 200 ms, respectively). Post hoc tests for duration revealed that overall integration at the durations from 30 ms to 70 ms significantly differed from overall integration at all other durations (all *p*s < .005 and all *d*s > 0.63). However, integration at 90 ms was only significantly higher than at 150 ms, *t*(28) = 3.661, *SE* = 0.013, *p* = .011, *d* = 0.364, at 180 ms, *t*(28) = 4.983, *SE* = 0.013, *p* < .001, *d* = 0.495, and at 200 ms, *t*(28) = 5.462, *SE* = 0.013, *p* < .001, *d* = 0.543, but not different from 110 ms and 130 ms. Furthermore, integration at 110 ms was significantly greater than at 180 ms, *t*(28) = 3.853, *SE* = 0.013, *p* < .001, *d* = 0.383, and 200 ms, *t*(28) = 4.331, *SE* = 0.013, *p* < .001, *d* = 0.431, while other comparisons did not reach significance, suggesting that overall integration performance was not affected much by changes in longer durations, particularly after 130 ms.

Planned pair-wise comparisons of integration performance between object-absent and out-object were significant at all durations (all *p*s < .001 and *d*s > 0.83), except for 180 ms and 200 ms. Integration between in-object and out-object also differed significantly at all durations (all *p*s < .004 and *d*s > 0.43). Lastly, integration in the object-absent condition was significantly higher than in the out-object condition at all durations (all *p*s < .01 and *d*s > 0.48), except for 150 ms and 200 ms. This suggested that the object effect on integration could start for stimuli as short as 30 ms and could last for those up to 150 ms.

The results thus showed that integration performance over time was in line with the long-standing finding that as the duration of the first stimulus is increased, integration performance declines until it reaches a critical point (approximately 100–150 ms), and beyond that point, further decreases in performance become insignificant or nonexistent (e.g., Di Lollo, 1977; Efron, 1973; Hogben & Di Lollo, 1974). The exact moment at which this occurs varies with stimulus conditions (e.g., luminance), and we only note that ours seems to fall within the commonly observed range without clear shifts due to the object conditions. Although in our task duration and object condition interacted statistically, the object effect seemed quite stable overall. Furthermore, the presence of an object affected integration even when the overall level of integration was already low, with only slight hints of it being affected by this bottom level.

## Experiment 3

Experiment 3 was carried out to investigate and compare the effects of object-based attention on temporal integration and segregation. Although integration and segregation in the MET might be considered two sides of the same coin, it is nevertheless conceivable that attentional modulation may impact them differently. For instance, as it has been shown that attention can increase perceived duration (Mattes & Ulrich, 1998; Yeshurun & Marom, 2008), this might negatively, rather than positively, impact temporal segregation.

### Method

#### Participants

Thirty-eight new students (12 males) took part in this experiment, and data from seven participants were excluded from the analysis because of poor performance. The average age was 22.42 years (range: 18–35).

#### Apparatus, stimuli, procedure, and design

**Fig. 5:**
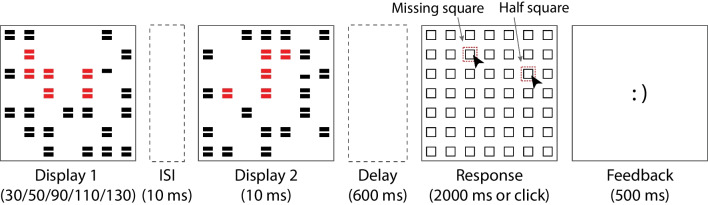
Schematic representation of a single trial used in Experiment 3. Two successive arrays of small black squares with a horizontal line gap in the middle were shown. Across two stimulus displays, one square was split in half, such that both displays showed one of two complementing half squares, and the location of one square in the grid was left empty. Thus, each trial contained both a missing location and a location in which only one half of a square was shown on each display. Depending on the instruction either the missing location or the location of the half squares needed to be located in the response screen. (Color figure online)

With the following exceptions, the setup of the experiment was the same as that of Experiment 1. In addition to measuring integration performance, segregation performance was tested. As shown in Fig. 5, next to the missing square, one other square in the array was split in half, such that both displays showed one of two complementing half squares. Thus, 47 out of 49 possible squares in the grid were filled with full squares across two displays, there was one missing location in the grid, which was the target for the integration task, and there was one split square location, which was the target for the segregation trials. All the object and duration conditions described above that applied to the integration target were the same for the segregation target. All square stimuli were presented with a horizontal line gap in the middle, in this way we aimed to reduce the sharpness of the contrast difference between the two halves of the segregation target when integrated. The first stimulus duration was 30, 50, 90, 110, or 130 ms. Integration and segregation tasks were performed in two separate blocks each. Instruction was provided at the beginning of the block to locate either the missing square (which required temporal integration) or the half-square (which required segregation). The order of the blocks was counterbalanced across participants. There were 1,680 trials that were divided into four blocks. Two-way repeated-measure ANOVAs were performed, with the factors duration (30, 50, 90, 110, and 130 ms) and object condition (in-object, out-object, and object-absent). Performance was analyzed for integration trials and segregation trials separately.

### Results

**Fig. 6:**
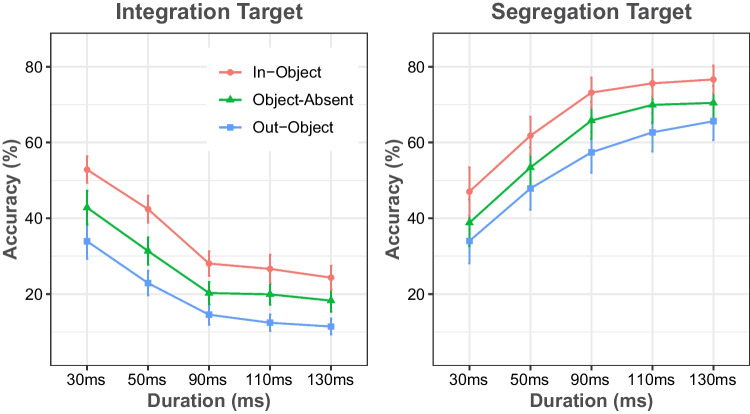
The percentage of correct responses for integration (left) and segregation (right) tasks in Experiment 3, plotted as a function of the duration of the first stimulus display, and separately for each object condition. Error bars represent 95% confidence intervals

Integration and segregation performance were analyzed separately with two-way repeated-measures ANOVAs. Figure 6a shows the integration rate in percentage. The analysis of integration yielded a main effect of duration, *F*(2.44, 73.193) = 180.69, *MSE* = 0.001, *p* < .001, η_p_^2^ = 0.858, and object,* F*(1.61, 48.33) = 194.924, *MSE* = 0.006, *p* < .001, η_p_^2^ = 0.867, as well as their interaction,* F*(5.784, 173.524) = 2.345, *MSE* = 0.005, *p* = .035, η_p_^2^ = 0.072. As in Experiment 2, it was observed that the integration rates decreased notably until 90 ms (43.8%, 32.4%, and 21.1% at 30, 50, and 90 ms; all *p*s < .001, *d*s > 1.18), but stayed similar at longer durations (19.7% and 18% at 110 and 130 ms, respectively). As in previous experiments, the in-object condition had the highest integration rates (35.2%), the object-absent condition had the second highest integration rates (26.7%), and the out-of-object condition had the lowest integration rates (19.1%; all *p*s < .001, *d*s > 0.8). Planned pair-wise comparisons revealed higher integration rates for the in-object condition than for the out-object one (all *p*s < .001, *d*s > 1.35), and the object-absent condition (all *p*s < .05, *d*s > 0.64), at all durations. Similarly, comparisons between object-absent and out-object conditions were significant at all durations (all *p*s < .05, *d*s > 0.65).

The subsequent examination of segregation performance revealed a main effect of both duration, *F*(1.88, 56.644) = 132.899, *MSE* = 0.024, *p* < .001, η_p_^2^ = 0.816, and object condition,* F*(1.39, 41.685) = 117.919, *MSE* = 0.008, *p* < .001, η_p_^2^ = 0.797, but the interaction of duration and object was only marginally significant, *F*(8, 240) = 1.904, *MSE* = 0.002, *p* = .06, η_p_^2^ = 0.06. As opposed to integration, the segregation rate increased with the increase of the first stimulus duration, which is also a typical pattern for segregation performance in MET. The accuracy for the segregation target was 40.6%, 54.8%, 66%, 69.7%, and 71.1% for durations of 30, 50, 90, 110, and 130 ms, respectively. The post hoc tests revealed that all durations significantly differed from each other (all *p*s < .005, *d*s > 0.25), except for the difference between 110 ms and 130 ms (*p* = .77). Similar to integration performance, the in-object condition (67.3%) had a higher segregation rate compared with the object-absent, (60.1%), *t*(30) = 9.688, *SE* = 0.007, *p* < .001, *d* = 0.497, and out-object conditions, (54%), *t*(30) = 11.912, *SE* = 0.011, *p* < .001, *d* = 0.913. The difference between object-absent and out-object conditions was also significant, *t*(30) = 9.025, *SE* = 0.007, *p* < .001, *d* = 0.416. Furthermore, planned pair-wise comparisons showed that segregation performance was higher for the in-object condition than for the out-object (all *p*s < .001, *d*s > 0.75), and no-object conditions (all *p*s < .003, *d*s > 0.39), at all durations. In addition, performance in the object-absent condition differed marginally from the out-object condition at 30 ms duration (*p* = .054), but was significantly higher at all other durations (all *p*s < .05, *d*s > 0.32).

The outcome of Experiment 3 showed that the object had a similar effect on both integration and segregation performance, despite the overall performance being higher in the segregation task than in the integration task. In both tasks, the target was detected best inside the object, worse in object-absent grids, and worst outside the object.

## Discussion

The three experiments presented here yielded similar results: Temporal processing of visual information was influenced by the presence of a spatial object, suggesting an interaction between the temporal and spatial organization of visual input. In particular, we found that the appearance of a simple object in the MET was able to improve integration performance, which could be mediated by the perceptual processing of, as well as the allocation of attention to, the object. This enhancement in integration was observed even in extremely brief stimulus presentations (as short as 30 ms). The object similarly increased performance on event segregation, despite its opposite perceptual requirements. In this, we observed no clear difference across different stimulus durations, such that there was no interaction between the rate of integration or segregation, and the magnitude of the object benefit.

In our experiments, when the missing element was present at a location within the object, we found increased integration performance (in-object vs. object-absent). However, integration performance decreased when the missing element was shown at a location outside the object (out-object vs. object-absent). This result is in agreement with the general finding that temporal integration can be affected by the deployment of spatial attention to the location of the critical stimuli, as we mentioned earlier. The question is what mechanism causes the object-related facilitation we observed. It has previously been suggested in some studies that enhanced integration with spatial attention originates from the distinct functions of magnocellular and parvocellular pathways during visual information processing (Yeshurun, 2004; Yeshurun & Levy, 2003). According to this idea, spatial attention facilitates parvocellular neurons while inhibiting adjacent magnocellular neurons. Parvocellular neurons not only have smaller receptive fields, higher spatial resolution, and higher color sensitivity, but they also have longer response durations, while magnocellular neurons are faster, more contrast sensitive, and have a higher temporal resolution (Derrington & Lennie, 1984; Maunsell et al., 1990; Merigan & Maunsell, 1993; Schiller & Logothetis, 1990; Solomon et al., 1999). Thus, the net effect of attention would be an increase in perceived duration, which would consequently improve integration. In our study, the salience of the object would draw attention to its location and increase spatial resolution there, but this would then also result in a drop in the temporal resolution and possibly the amount of time it takes for information to be integrated.

However, the fact that performance was also enhanced when the two stimuli had to be separated confounds this account of this effect because, according to this logic, segregation would be expected to decrease as perceived length increases with spatial attention. In fact, several pieces of evidence supported the hypothesis that temporal resolution tasks are degraded by exogenous attention (Hein et al., 2006; Rolke et al., 2008; Yeshurun, 2004; Yeshurun & Levy, 2003). For example, Yeshurun and Levy (2003) tested a two-flash fusion paradigm with exogenous cues in a series of experiments and found that exogenous cues impair the ability to detect small temporal gaps between stimuli that are presented after one another. As in our study, since the unexpected display of an object in a random location might also exogenously drive attention, the attention allocated to the object should impair segregation performance. The outcome of our third experiment revealed the opposite effect, showing that the object facilitates both segregation and integration performance in a similar way. This suggested that enhanced integration may not take place as a result of a trade-off between temporal and spatial resolution as a result of allocating spatial attention.

Other data also contradict the explanation that enhanced temporal integration occurs because spatial attention facilitates parvocellular neurons while inhibiting magnocellular neurons. For instance, in the color fusion study by Akyürek and van Asselt (2015), subjects were asked to specify which color they saw at a specific location, and both temporal integration and segregation performance were evaluated at the same time. Their results showed that color fusion improved with increased spatial attention by giving valid cues. However, since parvocellular cells are color sensitive while magnocellular are relatively color-blind (Merigan & Maunsell, 1993; Schiller & Logothetis, 1990), the color-reporting process in their task must have relied entirely on parvocellular neurons and magnocellular neurons should only have minimal impact on performance. Hence, Akyürek and van Asselt (2015) suggested that decreases in temporal resolution cannot be explained by the hypothesis that the impairment effect of attention arises due to the interaction of parvocellular and magnocellular channels, indicating that the underlying mechanism is likely to be different.

Furthermore, there is evidence that temporal resolution is not always negatively affected by exogenous attention (Nicol et al., 2009). It was shown that exogenous attention improves rather than degrades temporal resolution when response time is limited (Chica & Christie, 2009), or when the polarity of stimuli against the background was unmatched (Baek et al., 2007). Another line of evidence that supports the notion that attention can improve temporal resolution comes from studies in which attention was manipulated endogenously. Sharp and colleagues (Sharp et al., 2018) used a variant of the MET to examine how endogenous cueing to a location impacts integration as well as segregation. They found that both integration and segregation tasks were enhanced by using valid endogenous spatial cues, suggesting that spatial attention benefits both opposite temporal processes. This finding was interpreted as indicating that strategic attentional allocation based on endogenous cues modulates temporal processing in a flexible manner. There is also some neurological evidence to support this flexibility account; many studies have shown that alpha-band brain oscillations are associated with temporal integration and that events can be perceptually merged when they occur within the same oscillation cycle (Cecere et al., 2015; Milton & Pleydell-Pearce, 2016; Samaha & Postle, 2015; VanRullen, 2016; Wutz et al., 2014). It has been shown that alpha frequencies may increase or decrease depending on whether a task requires segregation or integration of visual information (Wutz et al., 2018). In line with this, a very recent study of Sharp and colleagues (Sharp et al., 2022) provided neurological evidence that attention modulation by a spatial cue benefits both temporal processes by altering the contralateral alpha frequency in the retinotopic visual cortex. More specifically, the contralateral alpha frequency was found to be faster than the ipsilateral alpha frequency in valid spatial cue trials when a task required the segregation of stimuli, but slower than the ipsilateral alpha frequency when the task required the integration of stimuli. In the present study, integration and segregation tasks were implemented in a block-wise manner, so it is possible that such alpha adaptation may have played a role here too.

When considering the possible role of spatial attention in our findings, it is important to note that attending to the object itself was not a task requirement in our experimental paradigm. Consequently, our results suggest that the object may have attracted attention independent of task-related strategies, given that performance in both tasks improved or degraded depending on the location of the missing element relative to the object. These results confirm some findings that attention can not only be deliberately directed toward a given goal, but that perceptual organization also drives attentional selection (Baylis & Driver, 1992; Driver & Baylis, 1989; Kramer & Jacobson, 1991). As mentioned previously, Kimchi et al. (2016) investigated how a perceptual object created according to various Gestalt factors can attract attention and found that the presence of an object positively affected task performance even though the object was not predictive or task related. Similarly, they found significant costs when the object and target did not share the same location and this cost grew as the target-object distance increased, which Kimchi et al. interpreted as evidence for the involvement of spatial attention. We observed a similar pattern of performance both during integration and segregation, suggesting that the allocation of attention to the object in our task may have provided advantages to detecting target items appearing at this location, while it may have caused disadvantages when target items appeared outside of the attended region.

While an account of our findings in terms of object-based attention is intuitively plausible, it must be noted that other processes involved in the perception of the object may also have played a role. The object was composed of squares in a different color, which defined contours in the stimulus arrays, upon which segmentation processes acted. The grouping of subcomponents, or the segmentation of an image are seen as the most important phases that enable object perception (Baylis & Driver 1992, 1993; Driver et al., 1992; Marr, 1982). Segmentation, which is frequently considered to occur early in visual perception, may in turn affect how attention is distributed in the scene. However, these intermediate perceptual processing steps could themselves also mediate the beneficial effects we observed on integration and segregation. Thus, we cannot determine to which extent the effects were driven by attention, by the “complete” object, and by these related processing steps.

Lastly, in both integration and segregation tasks, we observed the object benefit with stimulus durations as short as 30 ms; either improved performance when the target was at the object location or decreased performance when it was outside the object location. This may imply that perceptual objects or groupings (based on proximity and similarity of object elements) must have been perceived or processed very rapidly, because they modulated the integration and segregation processes that followed. If object detection took more time than allotted by this stimulus duration, then integration and segregation should have been modulated to a lesser extent than at longer durations, whereas we found that it was not. This idea is also compatible with earlier research demonstrating that Gestalt grouping happens preattentively (Duncan & Humphreys, 1989; Moore & Egeth, 1997). At the other end of the scale, considering the object effect on integration at longer stimulus durations, where the integration frequency is very low, performance did not change much beyond 130 ms. The finding that integration performance ceases to decline beyond this duration is typical (Di Lollo, 1977; Efron, 1973; Hogben & Di Lollo, 1974). However, the presence of an object had an impact on integration even after that duration, and object-based advantages on integration lasted up to 200 ms, when comparing in-object and out-object conditions. One explanation for this outcome may be that attention extends the perceived duration of the stimulus (Enns et al., 1999; Yeshurun & Marom, 2008) and may thereby expand the window for temporal integration (Megna et al., 2012). However, considering the arguments against this account presented above, an alternative interpretation might be that the localized processing of the object and its constituent features and/or the allocation of spatial attention causes a general perceptual sharpening, both in time and in space, allowing improved perception of any event, be it the absence of an element in the grid, or the presence of a half-square.

To conclude, we investigated the effect of object perception on temporal integration and integration in the MET. We discovered that presenting spatial objects in a temporal integration task can increase integration frequency, and that it can similarly affect segregation performance, which involves opposing temporal processes. This finding provides evidence for an interaction between spatial and temporal factors in the processing of visual input that seems to arise at the very beginning of the visual processing pathway.

## Supplementary Information

Below is the link to the electronic supplementary material.

Supplementary file1 (DOCX 35 KB)

Supplementary file2 (PDF 145 KB)

Supplementary file3 (PDF 143 KB)

## Data Availability

The datasets used in this study are available through the Open Science Framework repository, with the identifier 2kpn3 (https://osf.io/2kpn3/; 10.17605/OSF.IO/2KPN3)

## Code availability

The analysis scripts used in this study are available through the Open Science Framework repository, with the identifier 2kpn3 (https://osf.io/2kpn3/; htttps://doi.org/10.17605/OSF.IO/2KPN3)
