# Supplementary material for: The effect of object perception on event integration and segregation
Source: Atten Percept Psychophys. 2024 Sep 18;86(7):2424–37. doi: 10.3758/s13414-024-02922-6 (PMC11480121; doi:10.3758/s13414-024-02922-6)
Supplement: Supplementary file 1 — Supplementary file1 (DOCX 35 KB) [file 13414_2024_2922_MOESM1_ESM.docx]

**Supplementary Materials**

**Proximity to the center of the screen**

To assess the possible effect of the proximity of the target location to the center of the screen (i.e., at fixation) on the difference between the two object conditions, we ran an additional analysis in which we eliminated the five central target locations. Our reasoning was that the proximity effect would be most pronounced there, and much reduced at the remaining peripheral locations.

**Experiment 1**

The analysis revealed significant main effects of duration, *F*(1.85, 59.475) = 95.992, *MSE* = 0.007, *p* < 0.001, *η*_p_*²* = 0.75, and object, *F*(2, 64) = 65.771, *MSE* = 0.003, *p* < 0.001, *η*_p_*²* = 0.67, but their interaction was not significant. Accuracy averaged 37.8% at 50ms, 29.3% at 70ms, 25.8% at 90ms, and 22.9% at 110ms. Average accuracy for object conditions was 33 % in the in-object condition, 28.5% in the out-object condition, and 25.3% in the object-absent condition (see Figure S1a).

---Insert Figure S1 here---

Figure S1. The percentage of correct responses in Experiment 1 (left) and Experiment 2 (right),without trials on which the missing element appeared in the central five locations, plotted as a function of the duration of the first stimulus display, separately for each object condition. Error bars represent 95% confidence intervals.

**Experiment 2**

In Experiment 2, the main effects of duration, *F*(2.63, 76.328) = 71.311, *MSE* = 0.026, *p* < 0.001, *η*_p_*²* = 0.711, and object, *F*(2, 58) = 49.016, *MSE* = 0.009, *p* < 0.001, *η*_p_*²* = 0.628, were significant, whereas their interaction was marginally significant, *F*(8.93, 259.074) = 1.643, *MSE* = 0.007, *p* = 0.055, *η*_p_*²* = 0.054. Post-hoc tests revealed that overall accuracy was greater for the in-object condition (27.6%) than for the out-object (20%), *t*(28) = 9.867, SE = 0.008, *p* < 0.001, *d* = 0.581, and the object-absent conditions (23.3%), *t*(28)=5.645, SE = 0.008, *p* < 0.001, *d* = 0.332. There was also a significant difference between the object-absent condition and the out-object condition, *t*(28)=4.222, SE = 0.008, *p* < 0.001, *d* = 0.249. Post-hoc tests for duration revealed that overall integration at 30 ms (41.8%) and 50 ms (32%) significantly differed from overall integration at all other durations (all *p*s < 0.001 and all *d*s > 0.537). Integration at 70 ms (25%) was significantly higher than for all other durations (all *p*s < 0.05 and all *d*s > 0.22), except for 90 ms, and integration at 90 ms (22.1%) was only significantly higher than 180 ms (16.9%) and 200 ms (16.4%) (all *p*s < 0.008 and all *d*s > 0.397). Comparisons for integration at all other durations were not significant. Planned pair-wise comparisons of integration performance between in-object and object-absent were significant at all durations (all *p*s < 0.05 and *d*s > 0.526), except for 180 ms and 200 ms. However, the difference in integration performance between in-object and object-absent conditions was significant only at 30 ms (*p*s = 0.002 and all *d*s = 0.602), and all other comparisons did not reach significance (see Figure S1b).

**Experiment 3**

On integration, there was a main effect for both the object condition, *F*(2, 74) = 58.953, *MSE* = 0.006, *p* < 0.001, *η*_p_*²* = 0.614, and for the duration condition, *F*(1.93, 71.453) = 108.215, *MSE* = 0.017, *p* < 0.001, *η*_p_*²* = 0.745, while the interaction was not significant. Average integration was 33.8% at 30ms, 24% at 50ms, 14.9% at 90ms, 14.6% at 110ms and 12.8% at 130ms. Integration was 24.2% for the in-object condition, 19.4% for the object-absent condition, and 16.2% for the in-object condition (see Figure S2a).

The analysis of segregation revealed a main effect of duration, *F*(2.019, 74.692) = 149.076, *MSE* = 0.023, *p* < 0.001, *η*_p_*²* = 0.801, and of object, *F*(1.41, 52.04) = 156.560, *MSE* = 0.008, *p* < 0.001, *η*_p_*²* = 0.809, but their interaction was not significant. Average segregation performance was 66.6% in the in-object condition, 59.7% in the object-absent condition, and 53.3% in the out-object condition. Average segregation performance was highest at 130 ms with 70.6%, and then declined to 68.5%, 64.8%, 55.1%, and 40.4%, at 110ms, 90ms, 50ms and 30ms respectively (see Figure S2b).

---Insert Figure S2 here---

Figure S2. The percentage of correct responses for integration (left) and segregation (right) tasks in Experiment 3, without trials on which the missing element appeared in the central five locations, plotted as a function of the duration of the first stimulus display, and separately for each object condition. Error bars represent 95% confidence intervals.

Taken together, in all three experiments, overall performance in both integration and segregation was reduced compared to the analysis that included the center positions. This was expected since the target location appeared only in an off-fixation, more peripheral location in the present analysis, which is likely to impact performance negatively. However, both the overall pattern across time and the object effect remained consistent. Thus, the results suggest that the object effect was not solely due to the target positions inside the object being closer to the center.

**Error trials**

The tables below show where observers clicked on error trials, for each experiment. The mean values reflect locations near the missing element location (directly adjacent), at medium distances (1 square in-between), and at far distances (2 or more squares in-between).

**Table S1**

|  | | **Proximity** | | |
| --- | --- | --- | --- | --- |
| **Duration** | **Object** | Near | Medium | Far |
| 50 ms | In | 16.34 | 14.39 | 15.53 |
|  | Abs | 17.39 | 15.72 | 22.12 |
|  | Out | 17.16 | 18.16 | 18.44 |
| 70 ms | In | 17.41 | 17.94 | 18.62 |
|  | Abs | 18.22 | 18.11 | 28.03 |
|  | Out | 17.80 | 20.78 | 21.04 |
| 90 ms | In | 17.01 | 20.00 | 20.81 |
|  | Abs | 17.20 | 22.42 | 27.73 |
|  | Out | 16.78 | 22.35 | 22.44 |
| 130 ms | In | 19.26 | 21.38 | 21.67 |
|  | Abs | 17.31 | 22.80 | 30.08 |
|  | Out | 16.02 | 23.96 | 24.13 |

**Note.** Values show the percentage of trials in which responses landed at near, medium, and far distances from the missing element location in Experiment 1.

**Table S2**

|  | | **Proximity** | | |
| --- | --- | --- | --- | --- |
| **Duration** | **Object** | Near | Medium | Far |
| 30 ms | In | 17.02 | 12.14 | 11.25 |
|  | Abs | 18.45 | 15.83 | 16.91 |
|  | Out | 17.02 | 17.56 | 24.23 |
| 50 ms | In | 18.51 | 15.65 | 15.48 |
|  | Abs | 17.86 | 18.75 | 23.04 |
|  | Out | 17.32 | 22.44 | 30.30 |
| 70 ms | In | 21.07 | 20.30 | 17.44 |
|  | Abs | 15.95 | 21.79 | 27.86 |
|  | Out | 17.68 | 24.35 | 34.46 |
| 90 ms | In | 19.52 | 22.26 | 18.57 |
|  | Abs | 17.56 | 23.87 | 30.00 |
|  | Out | 17.14 | 23.99 | 38.27 |
| 110 ms | In | 20.36 | 22.08 | 19.94 |
|  | Abs | 19.11 | 23.04 | 30.66 |
|  | Out | 15.12 | 28.15 | 37.62 |
| 130 ms | In | 20.24 | 24.58 | 21.13 |
|  | Abs | 18.99 | 24.94 | 29.94 |
|  | Out | 14.94 | 25.00 | 41.31 |
| 150 ms | In | 21.55 | 24.46 | 22.26 |
|  | Abs | 17.98 | 26.25 | 32.26 |
|  | Out | 15.71 | 25.89 | 40.83 |
| 180 ms | In | 21.01 | 26.90 | 22.44 |
|  | Abs | 18.27 | 27.56 | 30.54 |
|  | Out | 14.88 | 28.51 | 41.19 |
| 200 ms | In | 22.74 | 26.49 | 23.57 |
|  | Abs | 17.08 | 27.14 | 32.62 |
|  | Out | 15.42 | 28.04 | 39.94 |

**Note.** Values show the percentage of trials in which responses landed at near, medium, and far distances from the missing element location in Experiment 2.

**Table S3**

| **Integration** | | | | |
| --- | --- | --- | --- | --- |
|  |  | **Proximity** | | |
| **Duration** | **Object** | Near | Medium | Far |
| 30 ms | In | 18.09 | 13.19 | 12.16 |
|  | Abs | 18.43 | 16.42 | 19.87 |
|  | Out | 16.36 | 19.41 | 27.88 |
| 50 ms | In | 18.38 | 17.97 | 18.66 |
|  | Abs | 18.61 | 19.70 | 28.80 |
|  | Out | 16.59 | 23.27 | 36.12 |
| 90 ms | In | 20.05 | 27.30 | 22.41 |
|  | Abs | 18.66 | 26.27 | 33.35 |
|  | Out | 15.78 | 28.11 | 40.50 |
| 110 ms | In | 20.51 | 28.17 | 22.81 |
|  | Abs | 18.03 | 26.67 | 33.76 |
|  | Out | 14.52 | 28.63 | 43.66 |
| 130 ms | In | 21.14 | 29.61 | 23.33 |
|  | Abs | 18.26 | 27.53 | 34.50 |
|  | Out | 13.36 | 28.05 | 45.79 |
| **Segregation** | | | | |
| 30 ms | In | 16.71 | 19.87 | 13.31 |
|  | Abs | 14.92 | 19.18 | 20.33 |
|  | Out | 11.06 | 18.49 | 24.54 |
| 50 ms | In | 11.81 | 16.01 | 13.08 |
|  | Abs | 10.48 | 15.96 | 17.17 |
|  | Out | 6.80 | 12.56 | 21.72 |
| 90 ms | In | 8.18 | 12.67 | 11.46 |
|  | Abs | 6.86 | 11.52 | 14.00 |
|  | Out | 4.90 | 10.54 | 18.43 |
| 110 ms | In | 7.20 | 10.71 | 10.66 |
|  | Abs | 5.36 | 10.20 | 12.73 |
|  | Out | 3.69 | 10.02 | 16.88 |
| 130 ms | In | 7.09 | 9.33 | 10.14 |
|  | Abs | 5.18 | 10.25 | 12.67 |
|  | Out | 5.36 | 7.20 | 14.34 |

**Note.** Values show the percentage of trials in which responses landed at near, medium, and far distances from the missing element location in Experiment 3.
