# Supplementary figures and images for: The effect of object perception on event integration and segregation
Source: Atten Percept Psychophys. 2024 Sep 18;86(7):2424–37. doi: 10.3758/s13414-024-02922-6 (PMC11480121; doi:10.3758/s13414-024-02922-6)

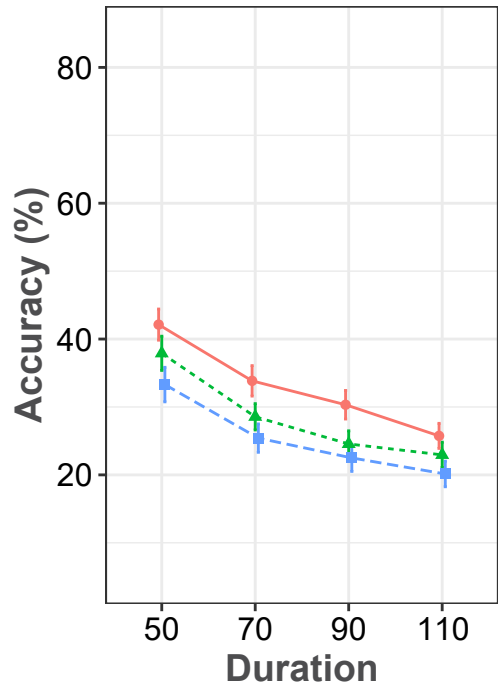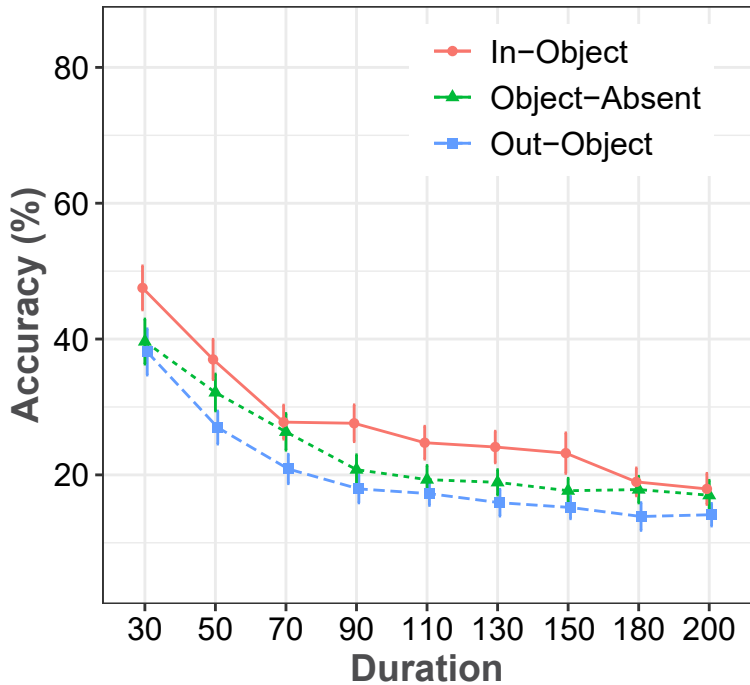

Supplement: Supplementary file 2 — Supplementary file2 (PDF 145 KB) [file 13414_2024_2922_MOESM2_ESM.pdf]

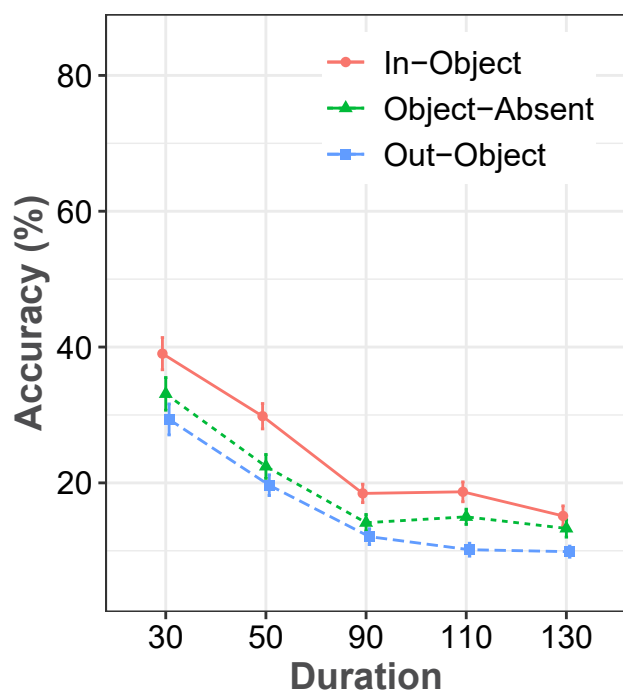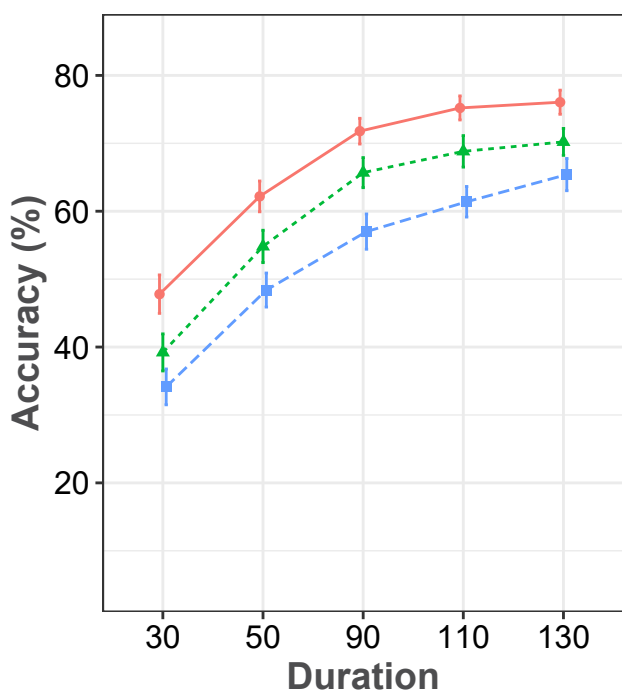

Supplement: Supplementary file 3 — Supplementary file3 (PDF 143 KB) [file 13414_2024_2922_MOESM3_ESM.pdf]
